# Helping Them Decide: A Scoping Review of Interventions Used to Help Minors Understand the Concept and Process of Assent

**DOI:** 10.3389/fped.2020.00025

**Published:** 2020-02-07

**Authors:** Pedro Weisleder

**Affiliations:** Division of Neurology and Center for Pediatric Bioethics, Nationwide Children's Hospital, The Ohio State University, Columbus, OH, United States

**Keywords:** research ethics, assent, dissent, pediatrics, human subject research

## Abstract

For adults, understanding research protocols prior to consenting to participate can be demanding. For children, that challenge is likely amplified. Yet, the participation of minors as research subjects is necessary. Otherwise, the likelihood of improving healthcare for minors now and in the future is hampered. The risk that minors could be harmed by procedures and medicines that are ill-adapted to their age-group or lack adequate scientific basis is real. It is therefore necessary to identify age-appropriate models to help minors understand the concept and process of assent. For this scoping review the concepts of assent and dissent, tools to evaluate the capacity of minors to assent, and six empirically based methods that have been used to help minors understand the process of assent were reviewed. Helping minors become better decision-makers in a manner that is commensurate with their development, supports children's prerogative to participate as human subjects in research.

## Introduction

The need to obtain consent prior to enrolment in a clinical trial is an obligation that has been included in guidelines which sanction human subject research since the beginning of the nineteenth century (1–4). These codes stipulate in one form or another that “Participation by individuals capable of giving informed consent as subjects in medical research must be voluntary, [and] no individual capable of giving informed consent may be enrolled in a research study unless he or she freely agrees” [(3), item 4 # 25].

The authors of the aforementioned documents recognized that certain individuals, or groups of individuals, are incapable of self-determination or have diminished autonomy (4). Their participation as human subjects requires added protections. Minors are one of the members of that category. From the explicit prohibition of their participation, to the requirement that others consent for them, the involvement of minors in research has at times been limited, and at times forbidden (1, 5). Setting limits to pediatric research deprives minors access to innovative treatments and other benefits of research. Disallowing the participation of minors in research trials increases their vulnerability (6).

## Methods

Herein is a scoping review on the concepts of assent and dissent, tools to evaluate the capacity of minors to assent, and six empirically based methods that have been used to help minors understand the process of assent. MEDLINE was searched using the terms: “assent,” and “minors as human research subject” alone or in combination. Papers that described empirically-based, structured methods to obtain minors' assent to participate as human research subjects were included in this review.

### Why Is the Participation of Minors in Human Subject Research Important?

A simplistic answer to this question is: because minors are not little adults. But reality is more complex.

- Research where minors participate as subjects is necessary to understand the physiological changes that the human body experiences from birth to adulthood (7).
- Research where minors participate as subjects is necessary to increase our understanding of normal and pathological development (7).
- Research where minors participate as subjects is necessary because the results of adult human subject research cannot be simply extrapolated to children, especially prepubertal ones (7).
- Research where minors participate as subjects is necessary as those with progressive, degenerative, and life-limiting conditions may not reach adulthood (7).
- Research where minors participate as subjects is necessary to increase our understanding of childhood illnesses and evaluate treatments and potential cures for those illnesses (8).
- Research where minors participate as subjects is necessary to establish therapeutic and harmful effects of medications. At this time, over 50% of drugs prescribed to minors have not been tested in their age group. In those instances, clinician have no alternative but to extrapolate to minors the doses of drugs used for adults (5, 9, 10).

In short, research where minors participate as subjects is necessary because without well-conducted research, there is no prospect of improving healthcare for minors now or in the future (8). As such, there is a real risk that minors could be harmed by procedures and medicines that are ill-adapted to their age-group (11).

### Assent

While the meaning of “consent” has been established and is internationally accepted, the same cannot be said of “assent” (6, 12). On the one hand, The Belmont Report states that “respect [for those whose comprehension is limited] requires giving them the opportunity to choose to the extent they are able, whether or not to participate in research” (4). Similarly, The Declaration of Helsinki merely states that in the case of individuals unable to give consent “the physician must seek [their] assent in addition to the consent of the legally authorized representative” [(3), 4 item #29]. On the other hand, 45 CFR 46.402(b) does offer a clear definition of assent “a child's affirmative agreement to participate in research” (13). Along those lines, Hein et al., define assent as the “affirmative agreement of a minor who is to take part in the informed consent procedure in a way adapted to his or her capabilities” (5). The point here is that an uncontested definition of assent is yet to be agreed upon (13).

If the definition of assent is yet to be solidified, regulations on the elements required to give informed assent are at best inconsistent, and at worse inexistent (13, 14). Researchers have indicated that currently available guidelines fail to address important elements including: age at which investigators should request assent; who should request assent; how much information should be shared with minors; how to resolve disputes between minors and parents; the relationship between assent and consent; and methods for assessing children's understanding the assent process itself (14). Several years ago, Bartholome (15) outlined four elements of valid assent:

1- A developmentally appropriate understanding of the nature of the condition.
2- Disclosure of the nature of the proposed intervention and what it will involve.
3- An assessment of the child's understanding of the information provided and the influences that impact the child's evaluation of the situation.
4- A solicitation of the child's expression of willingness to accept the intervention [(15), p. 4].

The American Academy of Pediatrics' Committee on Bioethics paraphrases Bartolome's elements of valid assent (16). The challenge with Bartholome's requirements is that empirical data to support their use is limited (17).

Adults are deemed to have decision-making capacity unless researchers have reasons to believe otherwise, or their circumstances place them in an at-risk category (i.e., prisoners). In the case of children, the assumption is reverse—they are presumed to lack decision-making capacity until proven otherwise (7). This belief leads to forms of unconscious bias. For example, researchers tend to judge a child to have decision-making capacity if the child's decision to assent conforms to their own ideas of what is in the child's best interest. Conversely, if the child seems to be making the *wrong* decision, adults take it upon themselves to guide the decision-making process to ensure the child makes the *right* decision (18).

To discern which minors can and which cannot assent to participate as human subjects, The Nuffield Council on Bioethics (7) proposes classifying minors into three categories:

1- Minors who are not able to contribute their own view as to whether they should take part in research, such as babies and very young children, or minors who are temporarily unable to contribute because they are unwell or unconscious.
2- Minors who are able to form views and express wishes, but are not yet able to make independent decisions about research involvement.
3- Minors and young people who potentially have the intellectual capacity and maturity to make their own decisions about taking part in a particular research study, but who are still considered to be minors in their domestic legal system.

As stated by the Council, the purpose of the classification system is not to provide simple answers to how minors at particular ages should be treated in clinical research, but to indicate three quite distinct situations in which a child's or young person's potential for input into a decision about research raises ethical questions, both for their parents and for professionals [(7), p. 101].

### Dissent

Merriam-Webster dictionary defines “dissent” as “to withhold assent or approval; to differ in opinion” (19). Germane to this paper, dissent means that minors can disagree to participate in a research trial, even in instances when consent for participation has already been given by a parent or guardian (8). Some guidelines which sanction human subject research, do not view dissent as an active endeavor. For example, the Belmont Report talks about “failure to consent,” or as is the case of children, “failure to assent.” The most recent revision of 45 CFR 46−2018—also treats dissent as passive “absent affirmative agreement” [(13), §46.402(b)].

The capacity to voice dissent has been tied to age, intelligence, maturity, level of education, accessibility to healthcare, socio-economic status, cultural background, native tongue, and gender (7, 12, 13, 20, 21). Young minors are less likely to dissent than adolescents because the former may not be aware that they can do so, while the latter may be willing to exercise their nascent autonomy (22). Accessibility to healthcare may influence a child's unwillingness to dissent (12). After all, participating in a research trial may mean that the child's health will be monitored frequently. Socio-economic status may influence the willingness of a child to dissent, especially in circumstances where compensation for participants may be viewed as trivial by the researchers, but significant by the child or her family (12). Cultural background and gender may exert an unstated influence, particularly in societies where children, especially girls, are not expected to question the decisions of adults. Finally, cultural background may also exert an unstated influence in societies where doctors are revered (23).

The concern over a child's ability to dissent garners additional importance when the child is asked to participate in non-beneficial research. Wendle indicated that minors should not be asked to participate in non-beneficial research that is more than minimally distressing (24). Given that in some instances distress may not be identified until it is experienced, he argues that a child should be given the opportunity to voice dissent regularly (24). Wendle's point is of paramount significance. Young minors have a proclivity to please, and fear disappointing. As such, they may be willing to endure a modicum of distress for fear of upsetting their parents. Similarly, they may be unwilling to voice objections for fear of disappointing a researcher who is also the child's doctor (13, 22).

Finally, some have questioned whether a child's dissent should be simply *considered*, or if it should be *respected* (18). Baines believes that, when it comes to dissent, we should stop paying lip-service to children. A child who is considered to have decision-making capacity should be allowed to assent. If the child is considered incapable of understanding the study, parents need to consent. The child may be involved in the deliberation, and parents should try to explain why they made the decision to consent, but at some point, a line needs to be drawn and a decision must be made by the parents or legal guardians (13, 18).

### Assessment of a Minor's Capacity to Assent

When minors participate in research trials, a parent or legal guardian is required to give consent, and as feasible, the child is to assent. In spite of the best efforts of researchers, Institutional Review Boards (IRB), and Research Ethics Committees (REC), if understanding research protocols can be challenging for adults, it is even more so for children (22, 25). To address this conundrum, researchers have devised tools to evaluate the capacity of minors to assent.

#### The MacArthur Competence Assessment Tool for Clinical Research(MacCAT-CR) (26)^1^

Researchers in clinical trials are responsible for ensuring that human subjects have the capacity to consent: understand the information presented; apply the information in a manner so as to be able to appreciate the subject's unique situation; and voice a consistent choice. In the case of individuals where the capacity for self-determination is in question, researchers need tools to evaluate if the prospective subject has the competence to consent. Of the different tools available, The MacCAT-CR has been determined to be the best competence assessment tool for adults (27). The MacCAT-CR is a semi-structured interview instrument for assessing decision-making capacity to consent to participation in human subject research (26). Researchers using The MacCAT-CR can adapt it to a wide range of studies and settings. The MacCAT-CR is designed to assess a subject's ability to: understand a research protocol; appreciate the effects of a decision about research participation; ability to reason or weigh risks and benefits; and ability to express a choice.

In most jurisdictions, individuals under 18 years of age cannot consent to participate in human subject research. To overcome this restriction, Hein et al., modified the MacCAT-CR to make it applicable to children (21). Their version of the MacCAT-CR has been determined to accurately assess children's competence to assent (21). Their conclusion is that in minors younger than 9.6 years of age, competence to assent is unlikely. In minors older than 11.2 years, competence to assent is probable. Minors aged 9.6–11.2 years are in transition, competence to assent may be justified when it can be demonstrated in individual cases.

### Interventions to Boost the Capacity of Minors to Assent

#### Personalized Assent

A child's capacity to assent should not depend exclusively on age. Other factors that play a role include: family dynamics; parents' willingness to afford autonomy to the child; the relationship between the child and the investigator; the child's experience with illness; the child's psychological state; the child's cognitive abilities; and the importance of the decision that needs to be made (6, 14). Taking these factors into consideration to tailor the assent process is called “personalized assent” (6). Personalized assent stands in contrast with the impersonal, age-based process.

#### Separate Consent Assent Sessions

Annett et al., consider that minors cannot dissent under social pressure (28). Such pressure can arise when a child is being asked to assent in front of her parents. This notion lead the authors to hypothesize that seeking assent from minors separate from their parents would be a more meaningful exercise. The researchers embedded their study inside a double blind, placebo-controlled trial comparing the effectiveness of two medications for asthma. Sixty-four child-parents units were randomized to learn about the asthma trial with or without their parents. Their results showed that minors that learned about the asthma trial apart from their parents asked more questions, were more engaged, and were better able to recall the information than those who learned about the trial in the customary way (28). The authors consider that the benefits they saw in the group of minors who learned about the trial apart from their parents were multifactorial: (1) all attention was placed on the child which in turn made her pay more attention to the conversation; (2) parental influences such as “hijacking the conversation” were not at play; (3) the ability to ask questions without fear of being judged; and 4- adolescents felt they were empowered to exercise self-determination (28).

#### Comic Books

Massetti et al., developed a comic book with the goal of helping minors understand the concept of assent using illustrations and simple language (29). Twenty minors between the ages of 7 and 12 years, and who were participating in a trial to evaluate performance on a computational task (AKA the *real study*), were enrolled. The comic book presented a short story with information on the *real study*. Information in the comic book included the invitation to participate in the *real study*, as well as the objectives, methods, instruments, procedures, risks, benefits, and the researchers' contact information. The comic book study's participants responded by using a Likert scale to score the clarity of the text and the illustrations. According to Massetti et al., most participants found the content of the comic book either “excellent” or “very good,” thus offering a viable option for obtaining informed assent (29).

#### Two-Step Decision Method

The two-step decision method requires that the IRB or ERC assess when to waive the requirement of assent (24). First, the IRB or ERC should determine whether the intervention in question offers participants the prospect of direct benefit. If such is the case, the assent of all minors who are capable of providing it should be required. If the intervention in question offers the prospect of direct benefit, but the intervention is only available to minors who participate in the study, the IRB or ERC may waive the requirement to obtain the participants' assent (24).

#### Educational Video

The Nuffield Council on Bioethics produced a cartoon video aimed at teaching minors about the importance of clinical research, and the process of assent (30). In the 3 min video, an animated version of a girl talks about the process that lead her and her parents to assent and consent, respectively, to participate in a clinical trial. Emphasis is placed on the importance of clinical trials, the right of the girl and her parents to ask as many questions as they want, the right to leave the trial at any time without consequences, and efforts to lessen the impact that participating in the trial may have on the girl's life. The video is available at: https://www.youtube.com/watch?v=6yaKwLG_vlE&feature=youtu.be.

#### Multimedia Approach

In 2011, O'Lonergan and Forster-Harwood reported the results of a study in which they combined visual and audio media—multimedia—to improve the comprehension of the process of assent. The multimedia tool was a subject-driven, PowerPoint (Microsoft Corporation, Redmond, WA) presentation which included hyperlinks to video content embedded in the presentation (25). The authors compared the subjects' comprehension of two medical procedures—abdominal ultrasound, and dual energy radiograph absorptiometry. O'Lonergan and Forster-Harwood reported that their tool was found to be more appealing, and yielded better understanding of the procedures than an IRB-approved assent form, a video with voice-over explanation, or an animated version of a boy reciting the voice-over explanation (25). The authors suggest that the multimedia approach is particularly well-suited for multi-center studies, as it standardizes the assent process.

## Discussion

For this paper I reviewed the concepts of assent and dissent, tools to evaluate the capacity of minors to assent, and six empirically-based methods that have been used to help minors understand the process of assent. Of the methods used to assess children's competence to assent, Hein et al.'s modification of the MacCAT-CR has the most empirical support (21). Of the methods used to help minors understand the process of assent, the comic book designed by Massetti et al., is best suited for children down to 7 years of age. The other methods are best suited for children 10 years and older.

## Conclusions

Research where minors participate as subjects is of paramount importance for all children's sake (8). Better comprehension of research protocols could lead to increased participation. Assent should be sought from minors who are deemed to have the capacity of doing so. Assent cannot be limited to ensuring that the regulatory box is checked off (16). Researchers should take advantage of validated tools to help minors understand the importance of clinical research, and the concept and process of assent.

## Author Contributions

The author confirms being the sole contributor of this work and has approved it for publication.

### Conflict of Interest

The author declares that the research was conducted in the absence of any commercial or financial relationships that could be construed as a potential conflict of interest.

## Footnotes

^1^Throughout their paper, Hein et al. use the term “competence” (21). In this section, I use term “competence” to remain true to Hein et al.'s text.
